# Occurrence, Sources, and Prioritization of Per- and Polyfluoroalkyl Substances (PFASs) in Drinking Water from Yangtze River Delta, China: Focusing on Emerging PFASs

**DOI:** 10.3390/molecules30112313

**Published:** 2025-05-25

**Authors:** Zixin Qian, Chao Feng, Yuhang Chen, Yuanjie Lin, Ziwei Liang, Hailei Qian, Jingxian Zhou, Jinjing Ma, Yue Jin, Dasheng Lu, Guoquan Wang, Ping Xiao, Zhijun Zhou

**Affiliations:** 1 School of Public Health, Fudan University, Shanghai 200032, China; 22211020168@m.fudan.edu.cn (Z.Q.); zjzhou@fudan.edu.cn (Z.Z.); 2 Shanghai Municipal Center for Disease Control and Prevention, State Environmental Protection Key Laboratory of Environmental Health Impact Assessment of Emerging Contaminants, Shanghai 200336, China; fengchao@scdc.sh.cn (C.F.); yuhangchen333@hotmail.com (Y.C.); linyuanjie@scdc.sh.cn (Y.L.); liangziwei@scdc.sh.cn (Z.L.); qianhailei@scdc.sh.cn (H.Q.); zhoujingxian@scdc.sh.cn (J.Z.); majinjing@scdc.sh.cn (J.M.); jinyue@scdc.sh.cn (Y.J.); wangguoquan@scdc.sh.cn (G.W.); xiaoping@scdc.sh.cn (P.X.)

**Keywords:** per- and polyfluoroalkyl substances, suspect screening, drinking water, prioritization, risk assessment

## Abstract

As regulations ban legacy PFASs, many emerging PFASs are being developed, leading to their release into the aquatic environment and drinking water. However, research studies on these emerging PFASs in drinking water are limited, and current standards only cover a few legacy PFASs, leaving many emerging PFASs unregulated and their toxicity unknown. Therefore, a machine learning-based suspect screening combined with target screening was employed to comprehensively identify and quantify both legacy and novel PFASs in drinking water from the Yangtze River Delta, and their potential sources of contamination were determined through pollutant profile analysis. A total of 30 PFASs were identified, including 16 legacy and 14 novel PFASs, categorized into 11 classes. Quantitative and semi-quantitative analyses revealed that the maximum concentrations of 30 PFASs ranged from <LOQ (limit of quantification) to 48.92 ng/L. Notably, PFPeA (48.92 ng/L), perfluorobutanoic acid (PFBA, 44.83 ng/L), perfluorooctanoic acid (PFOA, 37.72 ng/L), perfluorobutanesulfonic acid (PFBS, 26.77 ng/L), and bis(trifluoromethanesulfonyl)imide (HNTf2, 15.02 ng/L) exhibited higher concentrations compared to other PFASs. The pollutant profile analysis suggested that PFASs in the Yangtze River Delta’s drinking water are more likely to originate from pollution in the upper and middle reaches of the Yangtze River rather than from local industrial emissions. Then, the identified PFASs were prioritized by integrating the PBT (persistence, bioaccumulation, and toxicity) properties of PFASs with environmental exposure data. In the prioritization and risk assessment process, ten high-concern PFASs had Risk Indexes (RIs) higher than those of ref-PFOA and ref-PFOS, including eight legacy PFASs and two novel PFASs. The drinking water of the Yangtze River Delta originates from the surface water of the lower Yangtze River, which accumulates pollutants from its upper and middle reaches, affecting the health of over 20 million people. Our findings indicated the presence of emerging PFASs in the region’s drinking water and demonstrated conceptual models for integrating chemical information from suspect screening with toxicity prediction and risk assessment. Although the current levels of emerging PFASs are relatively low, legacy PFASs still dominate. Further research is needed to identify, monitor, and assess the health and environmental risks of emerging PFASs.

## 1. Introduction

Per- and polyfluoroalkyl substances (PFASs) are a class of anthropogenic compounds with high-energy of C-F bonds. Due to their hydrophobicity, lipophobicity, and high thermal and chemical stability, PFASs have been widely used in a variety of industrial and consumer applications [1]. Due to their widespread application and exceptional physicochemical properties, PFASs are ubiquitous in the environment [2]. Numerous epidemiological and toxicological studies have demonstrated that exposure to PFASs can cause a series of adverse effects on human health, including developmental and reproductive toxicity, neurotoxicity, hepatotoxicity, genotoxicity, immunotoxicity, endocrine toxicity, and carcinogenicity [3,4,5,6,7,8,9]. The persistence and toxicity of PFASs have raised global concern about their potential environmental and health risks. In order to reduce the contamination and health risks caused by PFASs, many countries and international organizations are implementing regulations to limit the manufacturing and utilization of some legacy PFASs, which has led to a growing number of manufacturers turning to the production of new alternatives of PFASs [10]. However, adequate safety tests are lacking for these novel PFASs, and companies refuse to disclose their components by citing trade secrets, making it difficult for regulatory agencies and the public to comprehensively assess their safety and long-term impacts [11]. Many of these novel PFASs are discharged into the environment and have been detected in both environmental and biological samples [10,12,13,14,15]. Previous studies have indicated that the toxicity of certain novel PFASs may be comparable to or even exceed that of legacy PFASs [16,17,18,19]. Many novel PFASs have shown higher affinity in molecular docking experiments with PPARα and ERα receptors than legacy PFASs [20].

Drinking water is one of the main pathways of human exposure to PFASs [21]. To safeguard public health, numerous countries and organizations have implemented or proposed standards to limit the presence of PFASs in drinking water. In 2024, the United States Environmental Protection Agency (EPA) established Maximum Contaminant Level (MCL) standards for six PFASs in drinking water. The specific standards are as follows: PFOA at 4 ng L^−1^, PFOS at 4 ng L^−1^, PFHxS at 10 ng L^−1^, PFNA at 10 ng L^−1^, and HFPO-DA (GenX) at 10 ng L^−1^. Additionally, the Hazard Index MCLs for mixtures containing two or more of the substances PFHxS, PFNA, HFPO-DA, and PFBS should be less than 1 [22]. Chinese sanitary standards for drinking water stipulated that the concentrations of PFOA and PFOS should not exceed 80 ng L^−1^ and 40 ng L^−1^, respectively. The current standards are primarily aimed at legacy PFASs and a small amount of novel PFASs. However, a significant portion of novel PFASs is not covered by existing standards of drinking water, and their toxicity remains unknown. Thus, comprehensively identifying and evaluating the risks of legacy and novel PFASs in drinking water is of utmost importance.

Many studies have demonstrated that target PFASs constitute only a minor portion of the total organic fluorine (TOF) present in living organisms and the environment. In fact, a significant portion of extractable organic fluorine (EOF) is attributed to unidentified PFASs [23]. EOF mass balance analysis revealed that target PFASs account for less than 36% of the EOF in drinking water from Shanghai [24]. This indicated the presence of a significant amount of unidentified organic fluorine compounds in the drinking water, underscoring the importance of identifying and studying these unknown pollutants. Currently, the majority of studies on PFASs in drinking water primarily focus on the routinely monitored targeted PFASs. However, the utilization of high-resolution mass spectrometry (HRMS) has enabled both suspect and non-targeted screening methods to identify unknown PFASs within complex environmental samples [11]. Only a few research studies have conducted suspect and non-targeted screening of PFASs in drinking water, but none of these studies have considered the toxicity and risk assessment of these emerging PFASs [24,25,26,27]. Linking the chemical information obtained from non-targeted screening to the toxicity of compounds is vital for risk assessment. This linkage is essential for identifying and evaluating potentially harmful substances that may not be regulated, thereby enhancing our ability to protect public health and the environment [28]. The Toxicological Prioritization Index (Toxpi) framework was designed to integrate multiple sources of information about exposure, compound properties, and health and environmental risk, enabling a comprehensive prioritization of chemicals based on their hazards to facilitate informed decision-making [29,30,31]. It was frequently utilized for risk assessment of multiple emerging pollutants in food, biological samples, and environmental media [12,32,33,34,35].

The Yangtze River Delta is located downstream of the Yangtze River. The local drinking water mainly relies on the surface water of the Yangtze River, which gathers various pollutants from the upper and middle reaches of the Yangtze River and is vulnerable to further contamination [36]. With the rapid urbanization and industrial development along the region of the Yangtze River, large amounts of industrial wastewater, domestic sewage, and agricultural runoff flow into the Yangtze River and its tributaries, resulting in a gradual deterioration of water quality [37]. Therefore, the safety of drinking water in the Yangtze River Delta has become particularly crucial. As a major economic center in China, the drinking water safety in the Yangtze River Delta directly affects the health of more than 20 million residents [24]. Ensuring the purity and safety of drinking water is not only an important measure to protect public health but also a key to maintaining regional economic stability and development.

In this study, a total of 49 pooled water samples were collected from the Yangtze River Delta. The aims of the study were to (1) comprehensively investigate the occurrence and concentrations of legacy and novel PFASs in drinking water samples through the combination of target and suspect analyses; (2) identify the potential sources of PFASs in drinking water of Yangtze River Delta by analyzing the contamination profiles of PFASs in drinking water, surface water adjacent to potential industrial emission sources in the local area, the Taihu Basin, and the Yangtze River; and (3) prioritize and risk assessment of identified PFASs according to their potential hazard effects and environmental exposure. The findings of our study would provide valuable insights into the prevalence of PFASs in drinking water from the Yangtze River Delta and the associated exposure risks among the general public.

## 2. Results and Discussion

### 2.1. Concentrations and Compositions of PFASs in Drinking Water

After preprocessing the raw data, we generated 414,397 peaks. Using suspect screening with blank subtraction and a retention time versus *m*/*z* filter, we identified 475 possible positive hits. Following the removal of duplicates and poorly shaped peaks, 88 peaks were selected for MS/MS spectra annotation. Through further structural elucidation based on diagnostic fragments, we ultimately identified 30 PFASs in the drinking water from the Yangtze River Delta. Among these thirty PFASs, fourteen legacy PFASs and seven novel PFASs were confirmed using authentic standards.

These 30 PFASs could be divided into 11 categories: (1) perfluoro carboxylic acid (PFCAs), (2) perfluoro sulfonic acid (PFSAs), (3) perfluoroalkyl dioic acids (PFdiOAs), (4) hydrogenated PFCAs (H-PFCAs), (5) hydrogenated PFSAs (H-PFSAs), (6) polyfluoroalkyl ether carboxylic acid(PFECAs), (7) fluorotelomer sulfonic acids (FTSAs), (8) chlorinated polyfluoroalkyl ether sulfonates (Cl-PFESAs), (9) hydrogenated polyfluoroalkyl ether sulfonates (H-PFESAs), (10) perfluoroalkyl sulfonamide(PFSMs), and (11) HNTf2.

PFCAs and PFSAs: By employing both target and suspect screening approaches, a total of ten PFCA homologues (C3−C12) and six PFSA homologues (C3–C8) were identified. Fourteen of them were confirmed by authentic standards based on exact mass, retention time, and MS/MS spectra (level 1a). PFPrA and PFPrS were assigned to level 2b. In the MS/MS spectra of PFCAs, the neutral loss of CO_2_ (*m*/*z* 43.98983) and the fragments [C_n_F2_n+1_]^−^ were commonly detected. The structures of PFSAs were determined using characteristic fragments, such as *m*/*z* 79.95736 [SO_3_]^−^, *m*/*z* 82.96085 [SO_2_F]^−^, *m*/*z* 98.95577 [SO_3_F]^−^, and [C_n_F_2n+1_]^−^. Among all the legacy PFASs identified, the maximum concentrations, in descending order, are as follows:

PFHxA (48.92 ng L^−1^) > PFBA (44.83 ng L^−1^) > PFOA (37.22 ng L^−1^) > PFBS (26.77 ng L^−1^) > PFPeA (8.75 ng L^−1^) >  PFHpA (8.42 ng L^−1^) > PFNA (7.59 ng L^−1^) > PFPrA (6.45 ng L^−1^) > PFOS (6.10 ng L^−1^) > PFPrS (5.32 ng L^−1^) > PFDeA (4.40 ng L^−1^) > PFHxS (3.73 ng L^−1^) > PFUdA (2.25 ng L^−1^) > PFPeS (0.16 ng L^−1^) > PFHpS (0.14 ng L^−1^) > PFDoA (0.09 ng L^−1^) (Table 1). PFHxA and PFBS exhibited the highest detection frequencies and were detected in all water samples. The concentrations of PFOA and PFOS in drinking water of Shanghai remained lower than the current Chinese Standards for drinking water (PFOA:80 ng L^−1^, PFOS:40 ng L^−1^), but above the MCL set by U.S. EPA (PFOA:4 ng L^−1^, PFOS:4 ng L^−1^) [22].

PFdiOAs: One PFdiOA homolog (C5) was identified through suspect screening. In the MS/MS spectrum of this homologous series, a neutral loss of 108 Da, equivalent to the loss of (CO_2_)_2_HF, was observed. Additionally, the fragment [C_3_F_5_]^−^ was detected in the spectrum (Figure S12). PFGdiA was detected in nearly half of the samples (DF = 0.46), and its maximum concentration was semi-quantitatively determined to be 1.5 ng L^−1^/. PFdiOA homologues (C9–C15) were found in airborne particulate matter and serum samples in China [14,15]. At present, PFdiOA has not been reported in drinking water. Currently, specific studies on the toxicity of PFdiOA are relatively scarce, and more research is needed to determine its effects on health and the environment.

H-PFCAs and H-PFSAs: We tentatively identified three H-PFCAs(C4–C6) and one H-PFSAs (C4) through suspect screening. The structures of H-PFCAs were identified using the predominant fragments [M−CO_2_−HF]^−^, which indicate neutral losses of CO_2_ and HF. The identification of H-PFSAs structures was based on the characteristic fragments of *m*/*z* 79.95736 [SO_3_]^−^, *m*/*z* 82.96085 [SO_2_F]^−^, and *m*/*z* 98.95577 [SO_3_F]^−^, as well as fragments [C_n_F_2n−1_]^−^ (Figures S2, S3, S5, and S6). Among the four compounds, HPFLSA_i n = 4 had the highest detection frequency and concentration (DF = 0.87, max = 7.42 ng L^−1^), followed by HPFLCA_i n = 4 (DF = 0.87, max = 0.03 ng L^−1^), HPFLCA_i n = 5 (DF = 0.77, max = 0.01 ng L^−1^), and HPFLCA_i n = 6 (DF = 0.20, max = 0.01 ng L^−1^). At present, H-PFCAs and H-PFSAs have been detected in various environmental media, including land landfill leachate, rainwater, surface water, and airborne particulate matter [10,13,14,38].

PFECAs: A total of three PFECAs(C3,4,6) were identified in our study. HFPO-DA was confirmed by authentic standards (level 1a), and the other two PFECAs were identified as level 2a. For this homologue series, neutral loss of CO_2_ (*m*/*z* 43.98983) and fragments [C_n_F_2n+1_O]^−^ were observed in the MS/MS spectrum (Figure S1). For these compounds, HFPO-DA had the highest detection frequency and concentration (DF = 0.79, max = 3.20 ng L^−1^), followed by PFMOAA (DF = 0.10, max = 0.43 ng L^−1^) and PFMPA (DF = 0.04, max = 0.81 ng L^−1^). HFPO-DA has been extensively utilized as a replacement for PFOA in various applications, including as a processing aid in fluoropolymer production and precursor for the synthesis of other fluorochemical compounds [39,40]. A study compared the developmental toxicity of HFPO-DA and PFOA and found that they had different effects on zebrafish embryonic development, indicating distinct modes of action. Additionally, HFPO-DA exposure specifically affected lipid metabolism, the HPT axis, and neurodevelopment, suggesting that HFPO-DA may not be a safe alternative to PFOA [39].

FTSAs: Two FTSAs, 6:2 FTSA and 4:2 FTSA, were identified through target screening. The characteristic fragments of this homologue series were *m*/*z* 79.95736 [SO_3_]^−^, *m*/*z* 82.96085 [SO_2_F]^−^, and [M-HF]^−^, which were observed in the MS/MS spectrum (Figure S8). 6:2 FTSA was detected in approximately 80% of the water samples, with the highest concentration reaching 8.17 ng L^−1^. 4:2 FTSA had a detection frequency of 0.2 and a maximum concentration of 0.75 ng L^−1^. 6:2 FTSA, as a replacement for PFOS, has been found to cause immunotoxicity in zebrafish embryos and hepatotoxicity in male mice according to studies [41,42]. These findings highlight the need for further investigation into the safety of 6:2 FTSA.

Cl-PFESAs: We identified one Cl-PFESAs (C8) by authentic standards in water samples. Structures of 6:2 Cl-PFESA were confirmed by fragments *m*/*z* 79.95736 [SO_3_]^−^, *m*/*z* 82.96085 [SO_2_F]^−^, and [C_6_ClF_12_O]^−^ (Figure S10). 6:2 Cl-PFESA was detected in five water samples (DF = 0.1), with the highest concentration reaching 0.18 ng L^−1^. 6:2 Cl-PFESA, as a substitute for PFOS, is widely used in multiple fields as a common fluorinated surfactant. Previous toxicological studies have indicated that 6:2 Cl-PFESA exhibits hepatotoxicity and endocrine toxicity, and its toxicity may be comparable to or even greater than that of PFOS [16,17,43].

H-PFESAs: One H-PFESA (C4) was confirmed by suspect screening and assigned to level 2a. The characteristic fragments of 2:2 PFESA were *m*/*z* 79.95736 [SO_3_]^−^, *m*/*z* 82.96085 [SO_2_F]^−^, [C_2_F_3_O]^−^, and [C_2_HF_4_O]^−^ (Figure S13). HPFESA_i n = 4 had a detection frequency of 0.12 and a maximum concentration of 0.75 ng L^−1^.

PFSMs: One PFSM(C6) was identified by suspect screening. Four characteristic fragments, including *m*/*z* 79.95736 [SO_3_]^−^, *m*/*z* 82.96085 [SO_2_F]^−^, [C_4_H_9_]^−^, and [C_4_HF_9_NO_2_S]^–^, were observed in the MS/MS spectrum of FBSAA and assigned to level 2b (Figure S7). PFSMs have many derivatives. The derivatives and homologues were previously detected in wastewater [44], landfill leachate [13], and surface water [12]. FBSAA was detected in approximately 24% of the water samples, with the highest concentration reaching 5.11 ng L^−1^.

HNTf2: The HNTf2 was identified by suspect screening and assigned to level 2b. Structures of HNTf2 were confirmed by three fragments *m*/*z* 79.95736 [SO_3_]^−^, *m*/*z* 82.96085 [SO_2_F]^−^, and [CF_3_SO_2_N]^−^ (Figure S4). It was detected in surface water in Beijing [12] and drinking water and landfill leachate in Shanghai [13,24]. This compound was widely used as a reagent, efficient catalyst, or additive in numerous reactions and was categorized as a dangerous chemical of Category 3 due to relatively notable acute toxicity in administered rats [12,45]. HNTf2 had the highest concentration among all emerging PFASs, with a detection frequency of 0.87 and a maximum concentration of 15.02 ng L^−1^. However, there is a lack of comprehensive studies on their toxicity.

In our study, there are three limitations: (1) We only used Oasis WAX cartridge to extract PFASs in water samples, and some PFASs not retained by Oasis WAX cartridge could not be analyzed; (2) For mass spectrometry analysis, we only selected the ESI (−) ionization mode, which may result in our results only including anionic PFASs and zwitterionic PFASs; (3) During the pre-treatment process, we found that some PFASs with 14–16 carbon atoms had low recovery rates (<10%), likely due to significant adsorption on the tube walls caused by their low polarity, which could affect the detection of certain long-chain PFASs.

### 2.2. Contamination Profile Analysis

**Figure 1:**
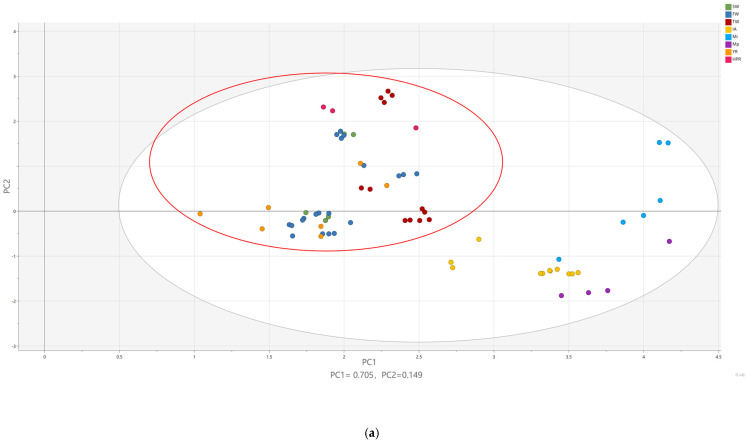

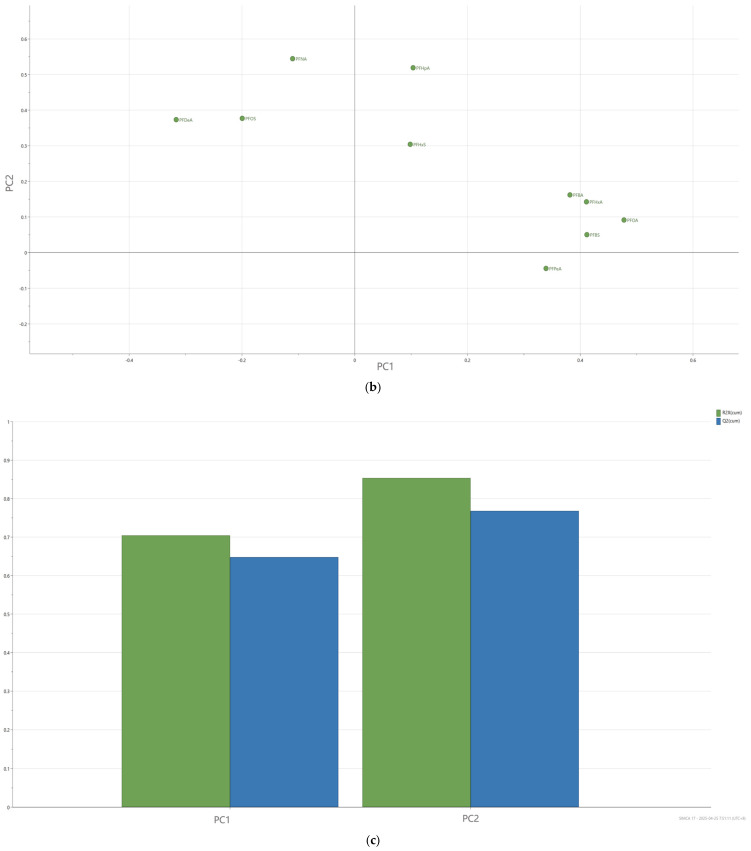
(**a**) PCA analysis of PFAS profiles in surface water from different sources. SW: source water; FW: factory water; TW: tap water samples; IA: international airport; Mi: industrial fluorochemical plant; Mp: metal plating plant; YR: Yangtze River; HPR: Huangpu River. (**b**) PCA loading plot. (**c**) Summary of the PCA model fit.

PFASs in drinking water could originate from different pathways. To further identify the potential sources of PFAS contamination in drinking water from the Yangtze River Delta, we collected data on the concentrations and types of PFASs in the Yangtze River and the Huangpu River and surface waters adjacent to potential industrial emissions around the Yangtze River Delta to conduct profile analyses [36,37]. The PCA results indicated that the profiles of our drinking water samples were completely separated from those of the surface water near Shanghai airport, fluorochemical plants, and metal plating factories, suggesting significant differences in profiles. In contrast, the profiles of our drinking water samples were overlapping with those of the surface water from the Yangtze River and the Huangpu River (Figure 1a, red circle). This suggested that the main source of PFAS contamination in drinking water from the Yangtze River Delta is more likely to be from upstream discharges into the Yangtze River rather than local industrial emissions. Principal Component 1 (PC1)explained 70.5% of the total variance, while Principal Component 2 (PC2) accounted for an additional 14.9%, resulting in a cumulative variance explanation of 85.4% (Figure 1a). The loading plot indicated that medium- and long-chain PFASs, such as PFNA and PFHpA, contributed significantly to (PC1) and were positioned in the upper part of the plot, suggesting their association with the variation along the (PC2) axis in the score plot. In contrast, short-chain PFASs, including PFOA, PFHxA, and PFBS, exhibited strong positive loadings on PC1, likely driving the separation of samples distributed on the right side of the score plot (Figure 1b and Figure S15). As shown in Figure 1c, the model’s goodness of fit was evaluated using R^2^X and Q^2^ (cum) values. The R^2^X values for the first and second components were 0.705 and 0.885 (cumulative), respectively, indicating a high proportion of the total variance was captured by the model. The Q^2^ (cum) value, derived from cross-validation, demonstrated the model’s predictive reliability. Since Q^2^ exceeded 0.5, the model was considered to possess good stability and predictive capability. These results suggest that the PCA model is suitable for further interpretation and analysis of group differentiation and variable contributions.

Among all the thirty identified PFASs, two novel PFASs, 6:2FTSA and HNTf2, had been reported in landfill leachate of Shanghai [13]. Notably, the concentration of 6:2FTSA reached as high as 69.2 ng/mL, suggesting that municipal waste management might be another significant source of novel PFASs in drinking water from the Yangtze River Delta.

### 2.3. Prioritization and Risk Assessment of Identified PFASs

**Figure 2:**
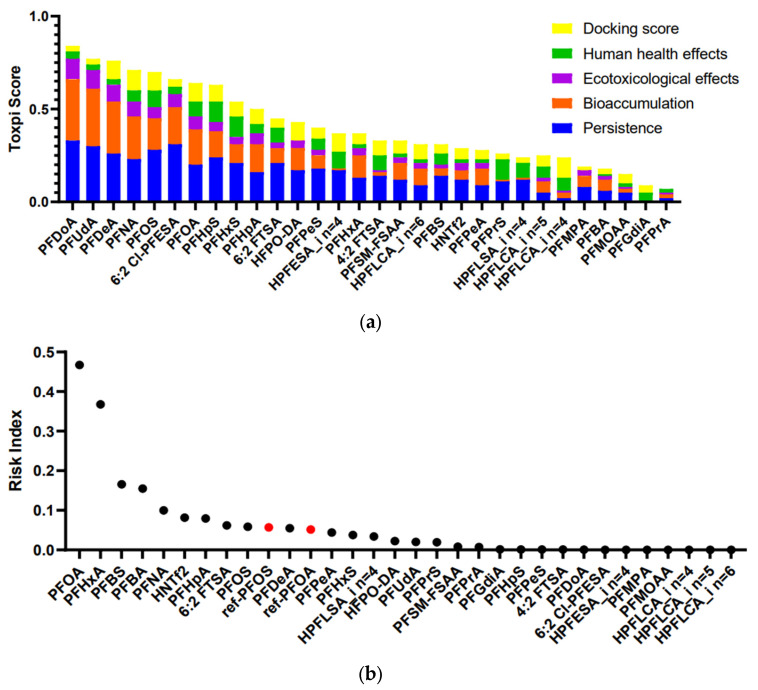
(**a**) ToxPi scores and (**b**) risk indexes of 30 PFASs identified and quantified in drinking water from the Yangtze River Delta. Black dots: identified compounds, red dots: reference points.

To prioritize the identified PFASs and pinpoint those of high concern, we utilized a risk-based prioritization approach based on the research by Hu et al. [12], with some modifications. Multiple hazard attributes were incorporated in our model, including persistence, bioaccumulation, and ecotoxicological effects, human health effects, molecular docking scores, and detection frequencies and concentrations. By integrating these multi-dimensional data, our model provided a comprehensive assessment of the potential environmental and health risks associated with the compounds, helping to prioritize those requiring immediate attention and regulatory action. Using PBT properties and molecular docking scores, the ToxPi scores calculated for the 30 identified PFASs ranged from 0.09 to 0.85 (Figure 2a), with PFDoA having the highest score, followed by PFUdA, PFDeA, and PFNA, all of which have higher scores than the traditional PFOA and PFOS. Generally, PFASs with longer carbon chains tend to have higher ToxPi scores. Among all the emerging PFASs, 6:2 Cl-PFESA has the highest ToxPi score (0.66), falling between the scores of PFOS (0.70) and PFOA (0.63). 6:2 Cl-PFESA, a proposed safe alternative for PFOS, may cause liver damage and induce lipid metabolism disorders in female mice through the action of PPAR-γ. Previous studies documented that the hepatotoxicity of PFOS and 6:2 Cl-PFESA appears to be higher than that of PFOA [17,46]. This indicated that we need to be mindful of the potential health and environmental hazards posed by these substitutes.

For persistence, the top 10 PFASs with the highest scores were PFDoA, 6:2 Cl-PFESA, PFUdA, PFOS, PFDeA, PFHpS, PFNA, 6:2 FTSA, PFHxS, and PFOA (Table S6). Higher persistence scores indicated that these compounds were less biodegradable in the environment and might pose long-term risks to environmental and human health. There was a total of eight PFASs with persistence scores higher than PFOS and PFOA. Notably, the two substitutes for PFOS, 6:2 FTSA and 6:2 Cl-PFESA, had high persistence scores. Among them, 6:2 Cl-PFESA ranked second in persistence scores, even surpassing its predecessor, PFOS. For bioaccumulation, we selected logKow (the logarithmic value of the octanol-water partition coefficient, indicating a compound’s hydrophobicity or hydrophilicity) and BAF (the ratio of a substance’s concentration in an organism to its concentration in the environment, reflecting its accumulation potential) as the key attributes to evaluate the accumulation potential and associated risks of compounds within organisms. Among all the identified PFASs, the ones with the higher bioaccumulation potential were PFDoA, PFUdA, PFDeA, PFNA, and 6:2 Cl-PFESA. These compounds exhibited higher bioaccumulation than both PFOA and PFOS (Table S6). In terms of ecotoxicity, the PFASs with higher toxicity scores were also PFDoA, PFUdA, PFDeA, PFNA, and 6:2 Cl-PFESA. We evaluated the human health effects of the compounds from five aspects: carcinogenicity, developmental toxicity, mutagenicity, skin sensitization, and oral LD50 in rats and endocrine toxicity. The PFASs with the highest comprehensive score, in order, were PFHxS, PFPrS, PFHpS, HPFESA_i n = 4, PFOS, 4:2 FTSA, HPFLSA_i n = 4, and PFOA (Table S6). We found eleven PFASs with possible carcinogenicity, two PFASs with potential developmental toxicity, and five PFASs with potential skin sensitization (Table S5). Based on the molecular docking scores with six receptors, the compounds with the highest total scores were PFNA, HPFLCA_i n = 4, HFPO-DA, PFDeA, HPFESA_i n = 4, PFOS, and PFOA (Table S6). Additionally, we found that some long-chain PFASs, such as PFDoA, PFUdA, and PFDeA, had lower docking scores compared to PFNA, likely due to limitations imposed by their molecular size.

Based on the measured detection frequency and concentrations, we utilized toxpi scores to calculate the RIs, which represented a synthesized assessment for prioritizing chemicals based on potential health risks. The continuous variables of PBT parameters, concentration, and detection frequency were converted into discrete ordered categorical variables (as described in Section 2.3 of the Methods), effectively reducing the influence of one or two outlier values on the final results. The RIs ranged from 0 to 0.54 for 30 PFASs in water samples (Figure 2b). PFOA had the highest RI, followed by PFHxA, PFBS, and PFBA. We found that some long-chain PFASs, such as PFDoA (Toxpi scores = 0.85, DF = 0.51, max = 0.09 ng L^−1^) and PFUdA (Toxpi scores = 0.77, DF = 0.57, max = 2.25 ng L^−1^), exhibited higher ToxPi scores and posed significant environmental and health hazards, and their RIs were not high when considering both detection frequencies and concentrations. Conversely, some short-chain PFASs, such as PFBA (Toxpi scores = 0.17, DF = 0.96, max = 44.83 ng L^−1^) and PFBS (Toxpi scores = 0.33, DF = 1, max = 26.77 ng L^−1^), despite having lower ToxPi scores, showed higher health risks due to their higher detection frequencies and concentrations (Table S6). To further identify the compounds of high concern, we selected the Maximum Contaminant Level (MCL) standard set by the EPA for PFOS and PFOA, which is 4 ng L^−1^, as a reference dose for risk assessment of the identified PFASs. A total of ten high-risk PFASs were identified through our method, including eight legacy PFASs and two emerging PFASs. Of these, PFOA had the highest RI (RI = 0.46), indicating it poses the highest risk. PFOA was closely followed by PFHxA, PFBS, and PFBA, suggesting that we need to be aware of the health risks posed by these short-chain PFASs. HNTf2 and 6:2 FTSA were the two emerging PFASs with the high risk. In the future, more research on their toxicity is necessary (Figure 2b).

## 3. Materials and Methods

### 3.1. Chemicals and Standards

Authentic standards of 68 target PFASs along with their 13 corresponding internal standards were purchased from Wellington Laboratories Inc. (Guelph, ON, Canada) for target analysis, and detailed information of these PFASs is provided in the Supporting Information (Table S1). The mixed standard solution and the mixed internal standard solution were prepared separately in methanol at 100 μg/L and stored at −20 °C. The ultrapure water was generated by a Milli Q system (18.2 Ω, TOC < 5 ppm, Merck, Bayswater, Australia), and HPLC-grade methanol and acetonitrile were purchased from Sigma-Aldrich (Castle Hill, NSW, Australia). In addition, all the other reagents (e.g., ammonium hydroxide, ammonium acetate) were purchased with HPLC grade from reliable suppliers.

### 3.2. Sample Collections and Pretreatment

Samples were collected from the Yangtze River Delta in August 2023. A total of forty-nine water samples were obtained. The sampling point map is shown in Supplementary Materials (Figure S14). High-density polyethylene (HDPE) bottles (1 L) and their caps were pre-rinsed using methanol (MeOH) and HPLC-grade water, then dried before use. The collected water samples were stored at 4 °C and extracted within 48 h using the routine solid-phase extraction (SPE) method.

Before extraction, 100 mL water samples were added with 0.925 g ammonium acetate (adjust PH to 6.8–7) and spiked with the internal standard solutions (20 μL of 100 μg/L each) before SPE. The extraction of water samples was performed by Oasis WAX Cartridges (150 mg, 6 mL, Waters, Milford, MA, USA). Briefly, the cartridges were preconditioned by 5 mL of 0.1% NH_4_OH in methanol, 5 mL of methanol, and 10 mL of Milli-Q water in sequence, then the water samples were passed through the cartridges at a flow rate of approximately 5–10 mL/min. After sample loading, the cartridges were rinsed with 5 mL 25 mmol/L ammonium acetate solution and 10 mL ultra-pure water and then dried under vacuum for about 15 min. The WAX cartridges were eluted with 2 mL of methanol and 4 mL of 0.1% NH_4_OH in methanol in succession. The eluents were nearly dried under a gentle stream of nitrogen and re-dissolved with 200 μL 60% methanol aqueous solution. After vortex mixing, the samples were centrifuged at a rotating speed of 16,000 RCF for 3 min. Then, the supernatants were collected into the sample bottle for analysis.

### 3.3. Instrumental Analysis

Target and suspect screening of the water samples was performed using an Agilent 1290 Infinity II LC system (Agilent, Santa Clara, CA, USA) coupled with an Orbitrap Exploris^TM^ 240 Mass Spectrometer (Thermo Scientific, San Jose, CA, USA) with electrospray ionization (ESI) source. Samples (5 μL) were injected onto an Agilent Infinity Lab Poroshell 120 EC-C18 analytical column (3.0 × 150 mm × 2.7 μm) with the column oven temperature set at 40 °C. Data acquisition was operated in negative ionization mode, utilizing both full scan (150−1500 Da) and data-dependent MS2 (ddMS2) scans to ensure comprehensive feature collection. The detailed parameters of chromatography and mass spectrometry can be found in the Supporting Information (Text S1).

### 3.4. PFAS Screening Workflow

**Figure 3:**
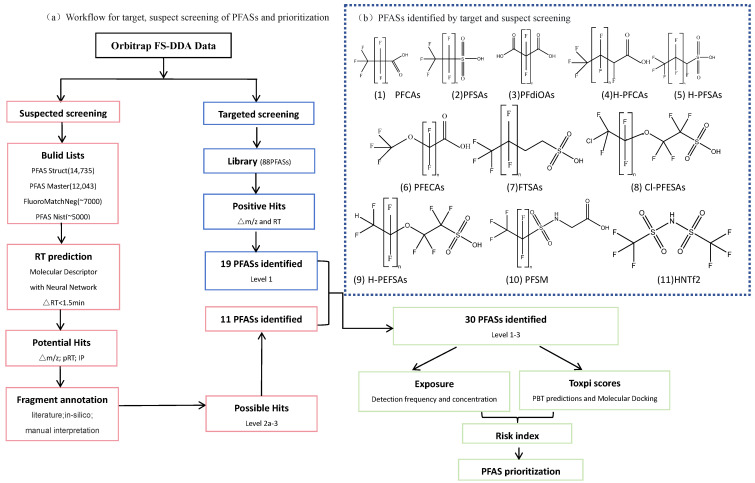
(**a**) Workflow for target and suspect screening of PFASs and prioritization and (**b**) proposed structures of PFASs identified by the target and suspect screening.

The suspect screening process (Figure 3) was conducted in accordance with our previous research [13]. An in-house library was established using 68 reference standards. This library was utilized for target screening and the development of a machine learning-based retention time (RT) prediction model. We collected four lists for suspect screening, including PFASSTRUCT v5 (14,735 PFASs, 785 cationic PFASs, as of August 2022) and PFASMASTER (12,043 PFASs, 817 cationic PFASs, as of August 2021) from the US EPA CompTox Chemistry Dashboard, FluoroMatch v3.3 (7206 PFASs, 493 cationic PFASs, accessed in September 2023), and “Suspect List of Possible PFAS” v1.7 (PFAS-Nist, 4967 PFASs, 539 cationic PFASs, DOI: 10.18434/mds2-2387, January 2023). These lists were compiled to build a comprehensive database, which was then screened using Compound Discoverer 3.3 (Thermo Scientific, Waltham, MA, USA). To improve data reliability and reduce the workload of subsequent manual annotation, specific filtering criteria were adopted to preliminarily eliminate low-quality and false-positive features. Key criteria for feature filtering in raw data included precise *m*/*z* values (<5 ppm), intensity >5 times the intensity in the extraction blank, IPs (fit threshold >70%, allowable intensity deviation <30%, and mass deviation <5 ppm), predicted retention time (<1.5 min), and identification of at least one characteristic fragment ion (<10 ppm). For structural confirmation, the formula-assigned features from suspect screening were further annotated by manual interpretation of their fragment ions or by comparing their MS2 spectra with the literature. Positive identification required that at least one characteristic fragment could be explained. The proposed structures were assigned three confidence levels (CLs) based on criteria established in Charbonnet et al.’s study [47]. Level 1 identification requires confirmation by matching a feature’s exact mass, isotope pattern, retention time, and MS/MS spectrum with existing standards. For Level 2 and Level 3 identification, the exact mass, isotope distribution pattern, predicted retention time, and characteristic fragments match those from databases and the literature reports, with at least two characteristic fragments matching. Level 3 lacks sufficient information to confirm a single structure, while Level 2 offers higher confidence in structural confirmation and can effectively distinguish isomers. 

### 3.5. Semi-Quantification of Suspect PFASs

In our study, a neural network model was applied to semi-quantify 11 PFASs without authentic standards by predicting their response factors (RFs), which are the slopes of concentration–response curves. The RF prediction model was constructed using 68 reference standards. A molecular descriptor set containing 3874 descriptors was generated from the 2D structures (canonical SMILES from PubChem) of these standards using alvaDesc v2.0. The RFs and molecular descriptor set of all 68 standards were randomly divided into training and test sets in an 8:2 ratio for model training to ensure objective evaluation and generalizability of the model [12]. The neural network algorithm, implemented in the Optuna mode (mljar-supervised v1.0, Python package), was employed to automatically tune the machine learning parameters. Over 5000 models were generated with various parameters, and the model with the best RMSE was selected as the optimized model. Using this optimized model, RFs for 10 PFASs without authentic standards were then predicted. The Python program we developed for predicting RFs was available on GitHub (https://github.com/SHCDC-LAB/the_prediction_model_for_RFs (accessed on 12 May 2025).

### 3.6. Quality Assurance/Quality Control (QA/QC)

A 100 mL volume of ultrapure water, spiked with the same internal standard solutions as the water samples, was used as a procedural blank to evaluate potential contamination during the extraction and analysis processes for each batch. The method LOD, LOQ, and RSD for 47 PFASs are detailed in the Supporting Information (Table S2). The calibration curves for each target compound exhibited high correlation coefficients (r^2^ > 0.99) (Table S2). Additionally, during the instrumental analysis, a standard mixture solution and methanol were sequentially injected following every six samples to act as calibration standards and blanks for data acquisition.

### 3.7. Molecular Docking

Results from animal experiments, in vitro tests, and epidemiological studies suggested that some PFASs can disrupt the endocrine system, interfering with the secretion of sex hormones and thyroid hormones and impacting normal reproductive ability, the nervous system, and immune function [48,49,50,51,52]. Receptor mediation is the primary mechanism by which compounds exert endocrine-disrupting activity [48]. Therefore, to assess the endocrine-disrupting effects of PFASs, six human hormone receptors (thyroid hormone receptors alpha (TRα, PDB ID: 3jzb) and beta (TRβ, PDB ID: 3gws), estrogen receptors alpha (ERα, PDB ID: 1ere) and beta (ERβ, PDB ID: 5toa), androgen receptor (AR, PDB ID: 3l3x) and peroxisome proliferators-activated receptors (PPARα, PDB ID:3vi8)) were selected for the receptor–ligand docking study. Molecular docking simulations were conducted using Biovia Discovery Studio 2021 software. The specific steps included protein and ligand preparation, protein–ligand docking, conformational analysis, and scoring function evaluation, following standard protocols implemented in the software. The specific processes were referred to in our previous research [13]. The Libdock scores were utilized to assess the binding affinity between the compound and the active site of the receptor, with higher scores indicating stronger affinity.

### 3.8. Prioritization and Risk Ranking of Identified PFASs

To prioritize the identified PFASs and pinpoint those of high concern, we utilized a prioritization approach that integrated data from multiple dimensions, including compound properties, ecological and health hazards, and environmental exposure. This approach was based on the research by Hu et al. [12], with some modifications. There were three steps in our method: (1) Firstly, we used quantitative structure–activity relationship (QSAR) models and molecular docking to predict the PBT properties of PFASs (Table 2 and Table S6). Based on these PBT properties, we calculated the ToxPi score for each PFAS using the ToxPi GUI software 2.0 (Equation (1)) [30]; (2) Secondly, we evaluated the exposure to compounds from two aspects: detection frequency and concentration, as outlined in Equations (2)–(4); (3) Ultimately, we calculated the risk index (RI) for each PFAS by combining its ToxPi score with the normalized exposure value through multiplication (Equation (5)) (Table S6. To further identify the compounds of high concern, we selected the Maximum Contaminant Level (MCL) standard set by the EPA for PFOS and PFOA, which is 4 ng L^−1^, as a reference dose for risk assessment of the identified PFASs.

Toxpi Score_i_ = W_p_ P + W_b_B + W_t_T (1)

Magnitude = (C_i_ − C_min_)/(C_max_ − C_min_) (2)

Exposure = DF × Magnitude(3)

Exposure_normalized_ = (Exposure_i_ − Exposure_min_)/(Exposure_max_ − Exposure_min_) (4)

RI = ToxPi Score × Exposure_normalized_ (5)

### 3.9. Data Analysis

Target and suspect analyses were performed using Trace Finder 5.0 software (Thermo Fisher Scientific, 2021, Waltham, MA, USA) and Compound Discoverer 3.3 software (Thermo Fisher Scientific, 2021, Waltham, MA, USA), respectively. The principal component analysis was conducted using SIMCA 17 software (Sartorius Stedim Data Analytics 2021, Umeå, Sweden). Logarithmic transformation, followed by ParN scaling, was applied to enhance comparability among variables.

## 4. Conclusions

In this study, target and suspect screening analysis using UPLC-Orbitrap HRMS was implemented to identify the occurrence and concentrations of legacy PFASs and novel PFASs in drinking water from the Yangtze River Delta. A total of 30 PFASs with high confidence levels (>3) were identified through both target and suspect screening, including 16 legacy PFASs and 14 novel PFASs. The pollutant profile analysis indicates that the PFASs in the drinking water of the Yangtze River Delta are more likely to originate from pollution in the upper and middle reaches of the Yangtze River rather than from local industrial emissions. By utilizing the ToxPi framework and RIs, we evaluated and prioritized the risks of identified PFASs in drinking water by integrating various data. A total of ten high-concern PFASs were identified through our method. While the priority ranking method used in this study provides valuable insights, it has certain limitations. The calculation of the RIs was based on a selected set of parameters, and the inclusion or exclusion of specific indicators may influence the final outcomes. It is also possible that some relevant factors were not incorporated into the current framework. Currently, legacy PFASs were the largest contributors to PFASs in the drinking water of the Yangtze River Delta, accounting for up to 83% of ΣPFASs, while the concentrations of emerging PFASs were relatively low. However, with some countries and organizations implementing regulations to ban the use of certain legacy PFASs, many companies are developing new alternatives to PFASs and introducing novel PFASs into the consumer market. This could result in increased concentrations of emerging PFASs in drinking water in the future. Therefore, more research studies are essential to comprehensively identify and monitor various emerging PFASs in drinking water and evaluate their potential health and environmental hazards.

## Figures and Tables

**Table 1:** Structure of PFASs identified at Level 3 or above.

Category	Structure/Proposed Structure	Acronym	N	CL	DF	Max (ng L^-1^)
PFCAs	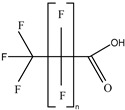	PFPrA	n 3	2b	63.27%	6.45
		PFBA	4	1	95.92%	44.83
		PFPeA	5	1	87.76%	8.75
		PFHxA	6	1	100.00%	48.92
		PFHpA	7	1	91.84%	8.42
		PFOA	8	1	97.96%	37.22
		PFNA	9	1	89.80%	7.59
		PFDeA	10	1	79.59%	4.40
		PFUdA	11	1	57.14%	2.25
		PFDoA	12	1	51.02%	0.09
PFSAs	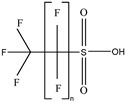	PFPrS	n 3	2b	69.39%	5.32
		PFBS	4	1	100.00%	26.77
		PFPeS	5	1	79.59%	0.16
		PFHxS	6	1	91.84%	3.73
		PFHpS	7	1	59.18%	0.14
		PFOS	8	1	67.35%	6.10
PFdiOAs	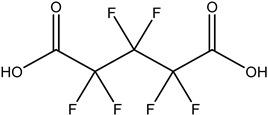	PFGdiA	n 5	1	46.94%	1.51
H-PFCAs	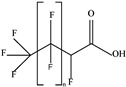	HPFLCA_i n = 4	n 4	3a	87.76%	0.03
		HPFLCA_i n = 5	5	3a	77.55%	0.01
		HPFLCA_i n = 6	6	3a	18.37%	0.00
H-PFSAs	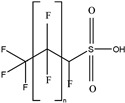	HPFLSA_i n = 4	n 4	2a	87.76%	7.42
PFECAs	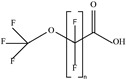	PFMOAA	n 3	2a	10.20%	0.43
		PFMPA	4	2a	4.08%	0.81
		HFPO-DA	6	1	79.59%	3.20
FTSAs	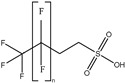	4:2 FTSA	n 6	1	20.41%	0.75
		6:2 FTSA	8	1	83.67%	8.17
Cl-PFESAs	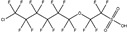	6:2 Cl-PFESA	n 8	1	10.20%	0.18
H-PEFSAs	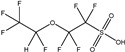	HPFESA_i n = 4	n 4	2a	12.24%	0.24
PFSM	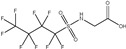	PFSM-FSAA	n 6	2b	24.49%	5.11
HNTf2	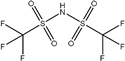	HNTf2	n 2	2b	87.76%	15.02

CL: confidence level; Max: maximum concentration; DF: detection frequency; N: number of carbon atoms.

**Table 2:** Details on the criteria and attributes used to calculate the ToxPi score of PFAS.

Criteria		Attributes		Unit	Scaling	Weight	Software	Model
Persistence		Biowin1		unitless	-x	1/9	EPI Suite v4.11	BIOWIN v4.10
		Biowin3		unitless	-x	1/9		BIOWIN v4.10
		Biowin5		unitless	-x	1/9		BIOWIN v4.10
Bioaccumulation		BAF		L/kg	log 10(x)	1/6	EPI Suite v4.11	BCFBAF v3.01
		Log *K_ow_*		unitless	x	1/6		KOWWIN v1.68
Toxicity	Ecotoxicological effects	Fish LC50 (96 h)		mg L^−1^	−log 10(x)	1/27	ECOSAR v2.0	ECOSAR v2.0
		Daphnid LC50 (48 h)		mg L^−1^	−log 10(x)	1/27		ECOSAR v2.0
		Green Algae EC50 (96 h)		mg L^−1^	−log 10(x)	1/27		ECOSAR v2.0
	Human health effects	Carcinogenicity		unitless	x	1/45	VEGA v1.2.0	CAESAR v2.1.10
								ISS v 1.0.3
								IRFMN-ISSCAN-CGX v1.0.1
								IRFMN-Antares v1.0.1
		Developmental toxicity		unitless	x	1/45	VEGA v1.2.0	CAESAR v2.1.8
							VEGA v1.2.0	PG v1.1.2
							T.E.S.T. v5.1.1	T.E.S.T. v5.1.1
							QSAR Toolbox v 4.5	DART
		Mutagenicity		unitless	x	1/45	VEGA v1.2.0	Consensus v1.0.4
								CORAL v1.0.1
								IRFMN-VERMEER v1.0.1
								IRFMN v1.0.2
		Skin sensitization		unitless	x	1/45	VEGA v1.2.0	CAESAR v2.1.7
								IRFMN-JRC v1.0.1
								NCSTOX v1.0.1
								TOXTREE v1.0.0
		Oral rat LD50		mg L^−1^	−log 10(x)	1/45	T.E.S.T. v5.1.1	T.E.S.T. v5.1.1
	Molecular docking	Endocrine disrupting effect	ERa	unitless	x	1/45	Biovia Discovery Studio 2021	Biovia Discovery Studio 2021
			ERb	unitless	x	1/45		
			AR	unitless	x	1/45		
			PPARa	unitless	x	1/45		
			TRa	unitless	x	1/45		
			TRb	unitless	x	1/45		

## Data Availability

The data presented in this study are available on request from the corresponding author.

## Supplementary Materials

The following supporting information can be downloaded at https://www.mdpi.com/article/10.3390/molecules30112313/s1, Figure S1: The MS2 spectra of PFMOAA. Figure S2: The MS2 spectra of HPFLCA_i n = 4. Figure S3: The MS2 spectra of HPFLCA_i n = 5. Figure S4: The MS2 spectra of HNTf2. Figure S5: The MS2 spectra of HPFLSA_i n = 4. Figure S6: The MS2 spectra of HPFLCA_i n = 6. Figure S7: The MS2 spectra of FBSAA. Figure S8: The MS2 spectra of 6:2 FTSA. Figure S9: The MS2 spectra of HOPFLSA n = 6. Figure S10: The MS2 spectra of 6:2 Cl-PFAES. Figure S11: The MS2 spectra of PFMPA. Figure S12: The MS2 spectra of PFGdiA. Figure S13: The MS2 spectra of HPFLSA_i n = 4. Figure S14: The Sampling point map. Figure S15: The VIP of variable in OPLS-DA. Table S1: Information of 68 reference standards and their 13 corresponding internal standards. Table S2: The recoveries and LOD, LOQ of 47 PFAS. Table S3: PFAS identified by suspect and nontarget screening with confidence level of 3 or above. Table S4: The concentrations of identified and confirmed PFASs in drinking water samples. Table S5: The PBT parameters used for calculating ToxPi. Table S6: ToxPi scores, exposure, and risk indexes (RIs) of PFAS in drinking water samples.
