# Supplementary material for: Occurrence, Sources, and Prioritization of Per- and Polyfluoroalkyl Substances (PFASs) in Drinking Water from Yangtze River Delta, China: Focusing on Emerging PFASs
Source: Molecules. 2025 May 25;30(11):2313. doi: 10.3390/molecules30112313 (PMC12155778; doi:10.3390/molecules30112313)
Supplement: Supplementary file 1 [file molecules-30-02313-s001.zip › supplementary material.pdf]

Text S1

2% Methanol aqueous solutions containing 5 mM ammonium formate and 100%methanol solutions were used as mobile phases A and B, respectively. The column was equilibrated with 5%mobile phase B for 3 min before injection. The gradient elution condition was as follows: 5%B (0.0−0.5 min), 5−99%B (0.5−11.0 min), 99%B (11.0−14.0 min), 99−5%B (14.0−14.1 min), and 5%B (14.1−15.0 min). The temperatures of the column oven and autosampler were set at 40 °C. The injection volume was 5 μL.

Q-Exactive (Thermo Scientific,Bremen,Germany) mass spectrometer,equipped with a heated electrospray ionization source, was operated in the negative mode and acquired the data using full scan (FS) with data dependent acquisition (DDA) mode (FS-DDA). The parameters of the source were as follows: spray voltage:−3.2 kV (ESI−)and +3.5 kV (ESI+); sheath gas flow rate:40 arb.unit; auxiliary gas flow rate:10 arb.unit; capillary temperature:320 °C; S-lens RF level:60; and auxiliary gas temperature:320 °C. Each FS-DDA loop contains one MS1 scan plus five MS2 scan. The resolution, automatic gain control, and maximum injection time (IT)in MS1scan (m/z 100−1000) were set at 70,000 fwhm (m/z 200), 1×106, and 100 ms,respectively; and they were separately set at 17,500 fwhm, 1 ×105, 50 ms in MS2 scan. Besides,the parameters including isolation window (2Da), normalized collision energies (NCE =20,40,60), apex trigger (2−5 s), exclude isotopes (ON), and dynamic exclusion (5 s)were set to get higher quality MS2 acquisitions.

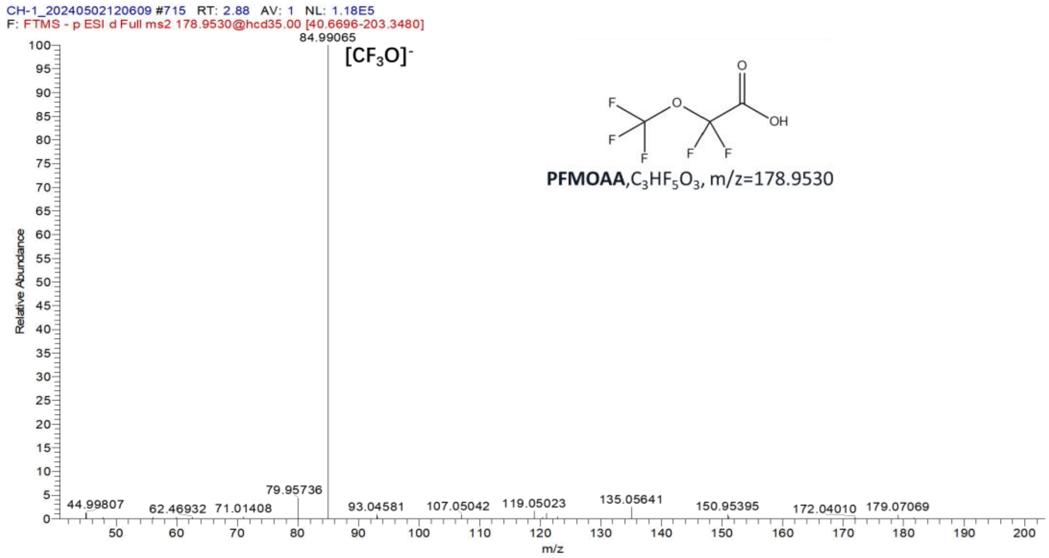

Figure S1 The MS2 spectra of PFMOAA

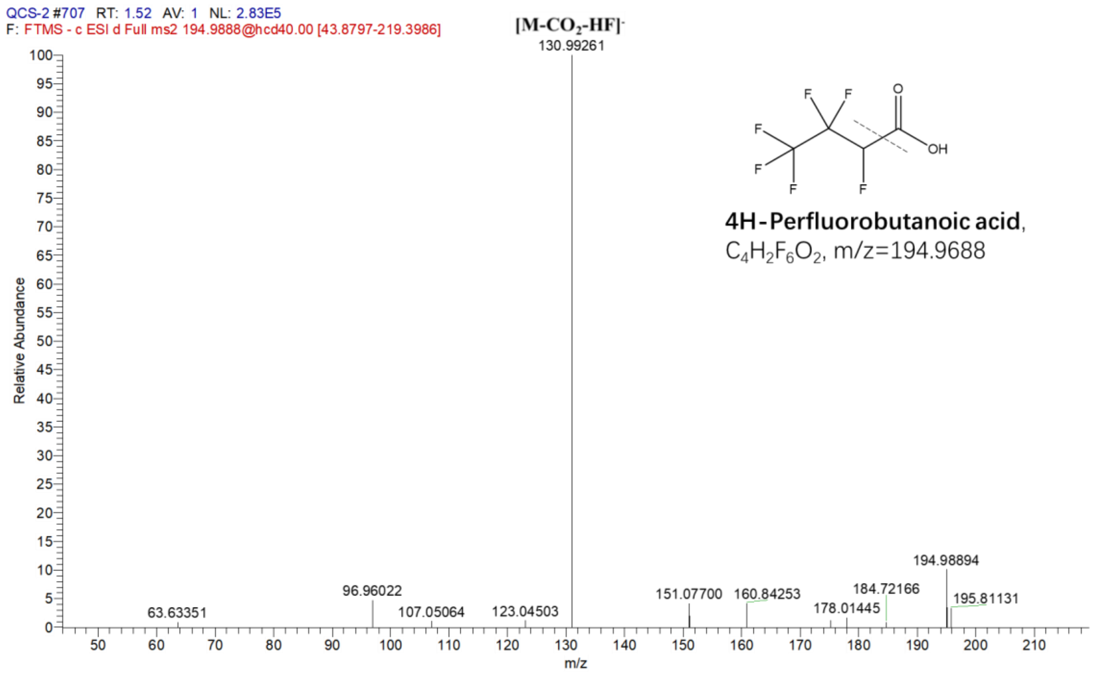

Figure S2 The MS2 spectra of HPFLCA_i n=4

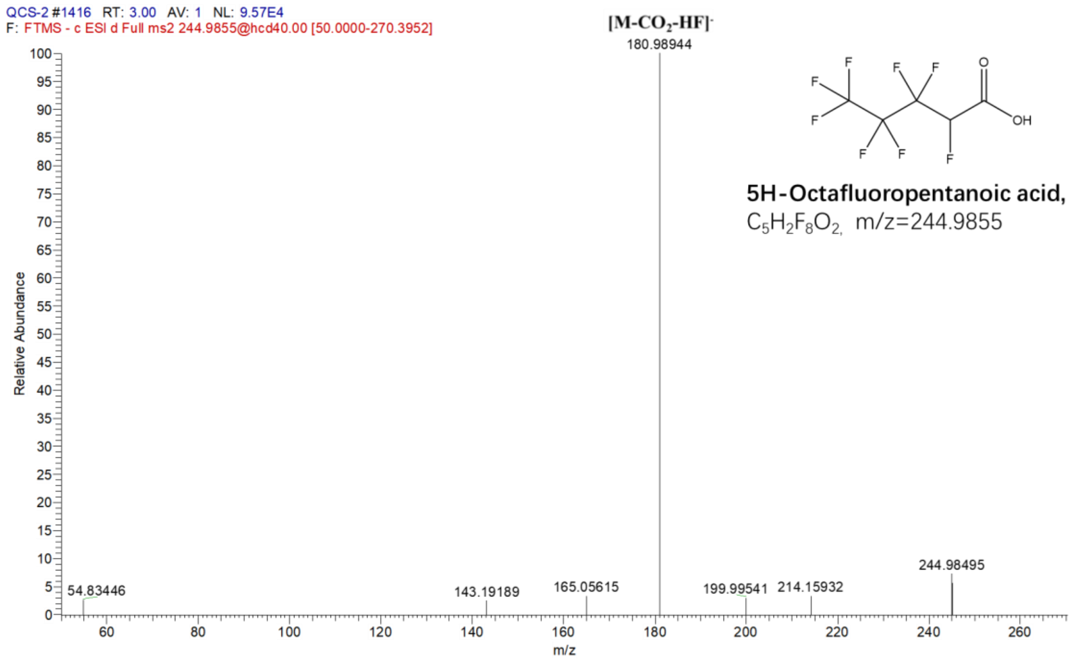

Figure S3 The MS2 spectra of HPFLCA_i n=5

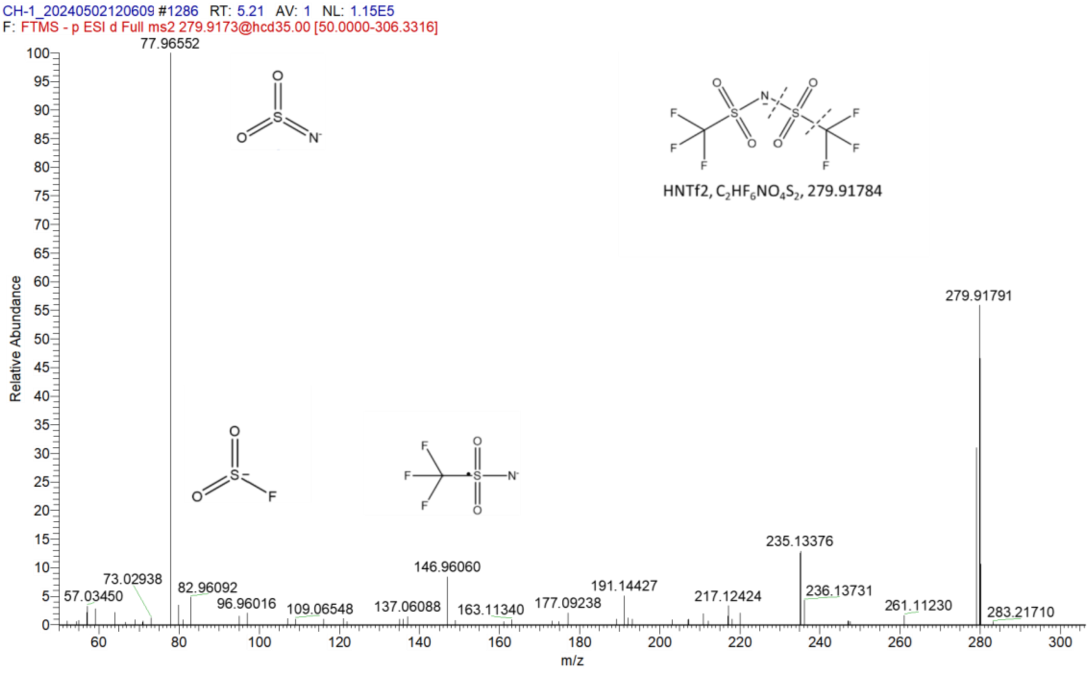

Figure S4 The MS2 spectra of HNTf2

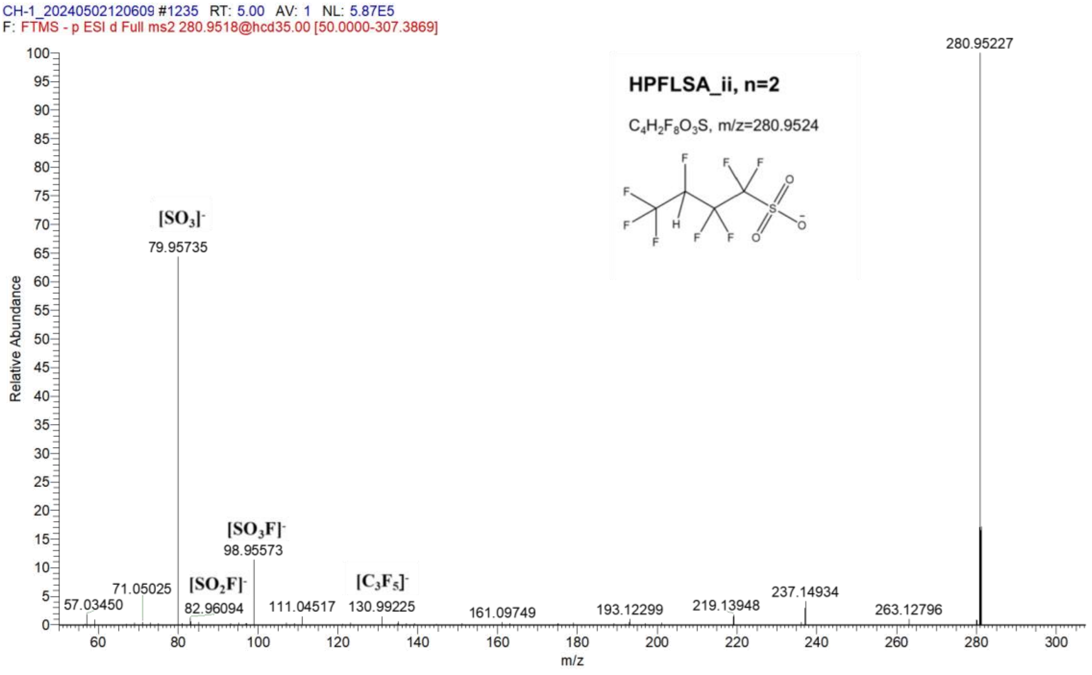

Figure S5 The MS2 spectra of HPFLSA_i n=4

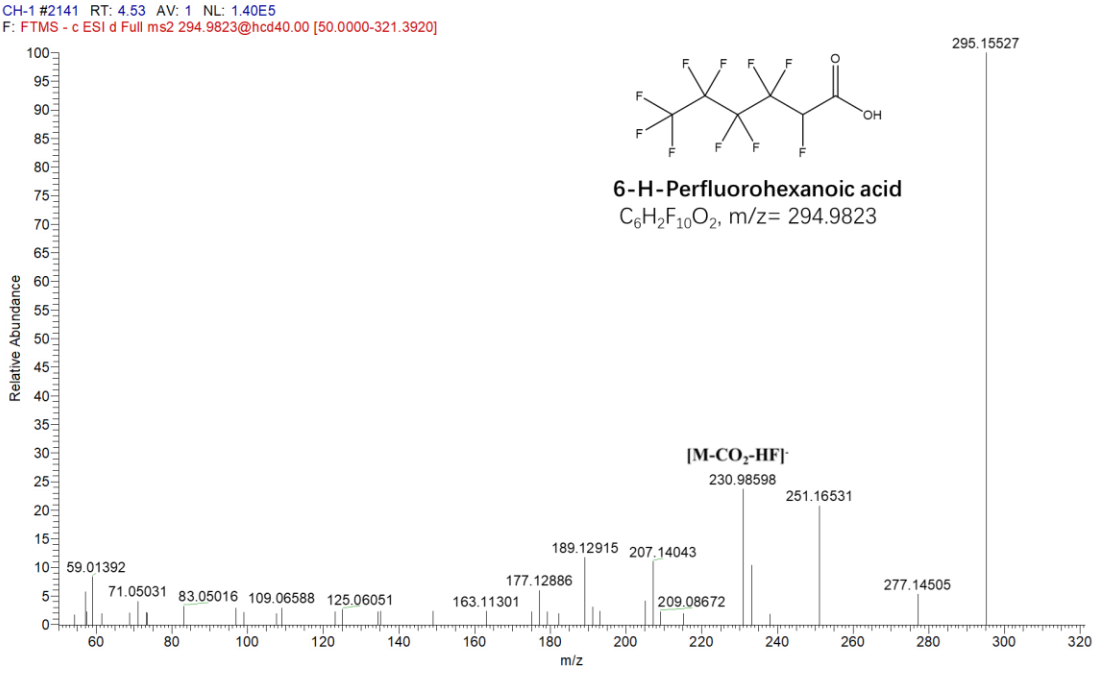

Figure S6 The MS2 spectra of HPFLCA_i n=6

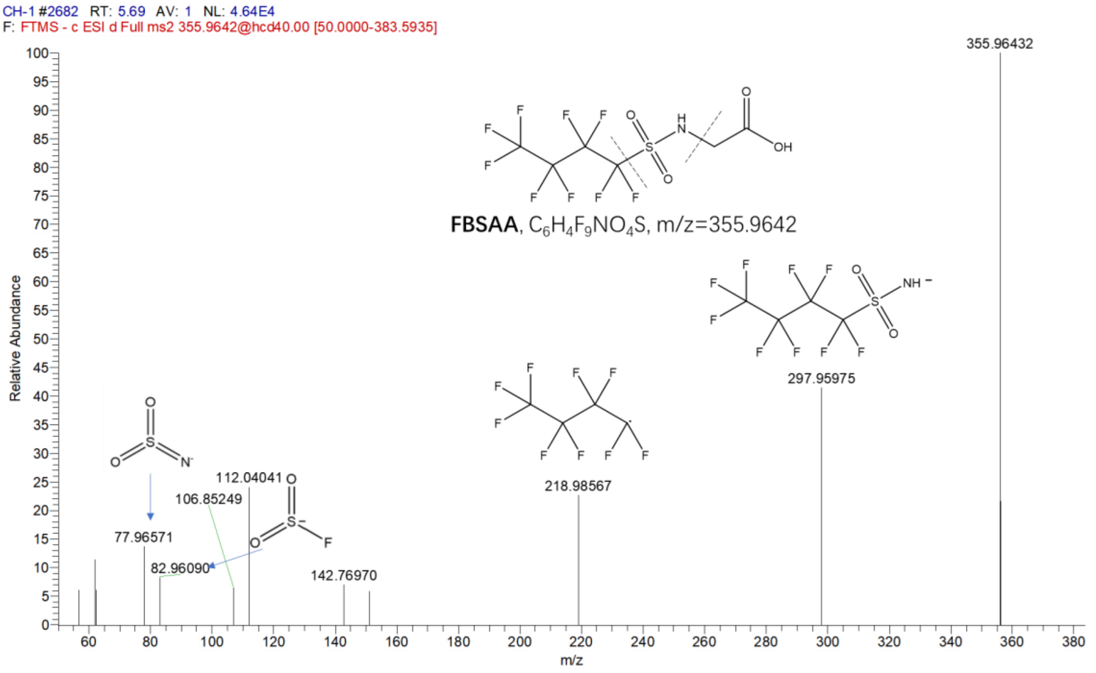

Figure S7 The MS2 spectra of FBSAA

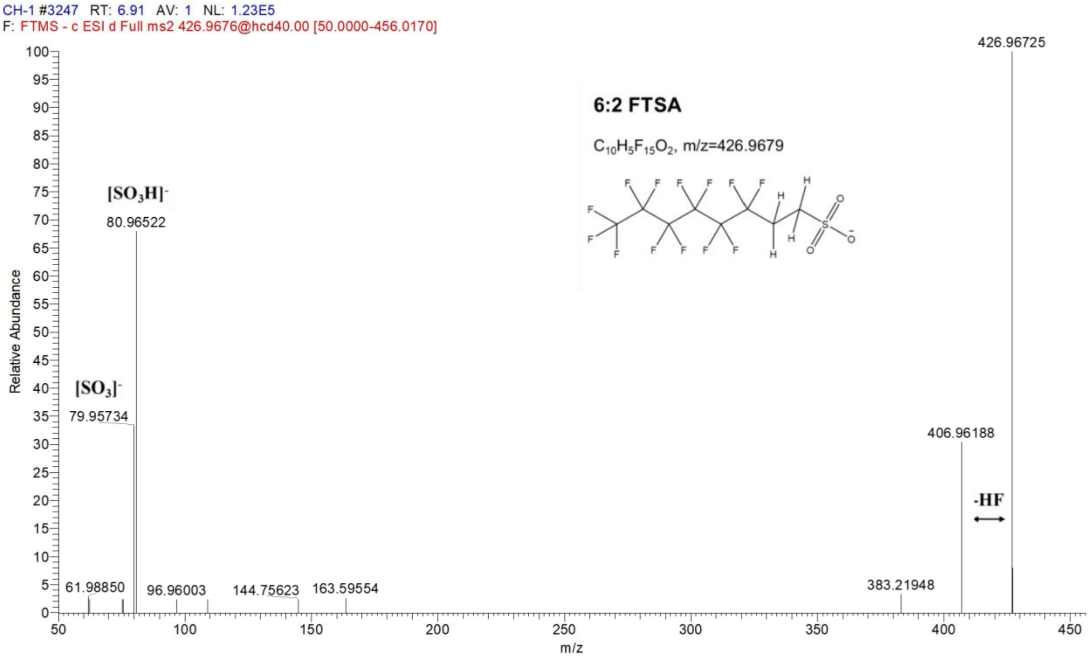

Figure S8 The MS2 spectra of 6:2 FTSA

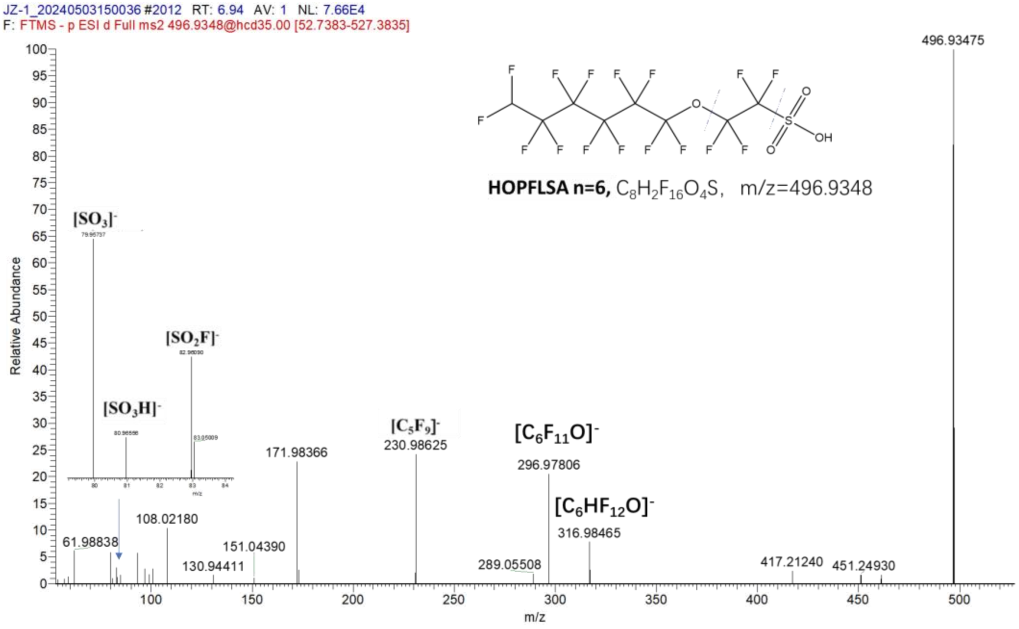

Figure S9 The MS2 spectra of HOPFLSA n=6

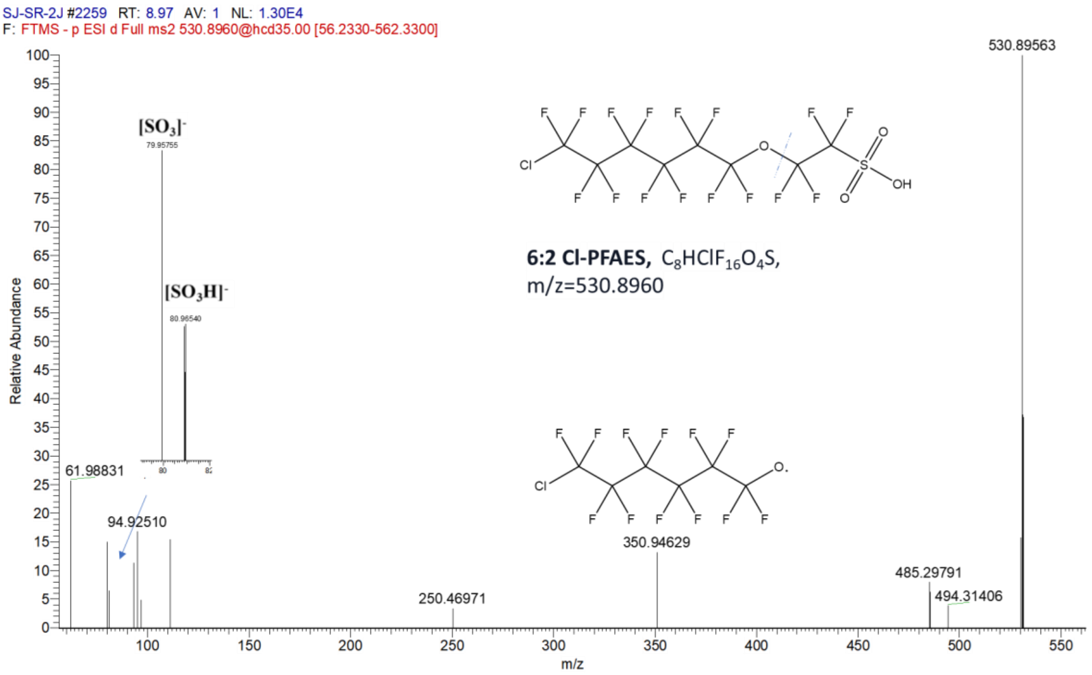

Figure S10 The MS2 spectra of 6:2 Cl-PFAES

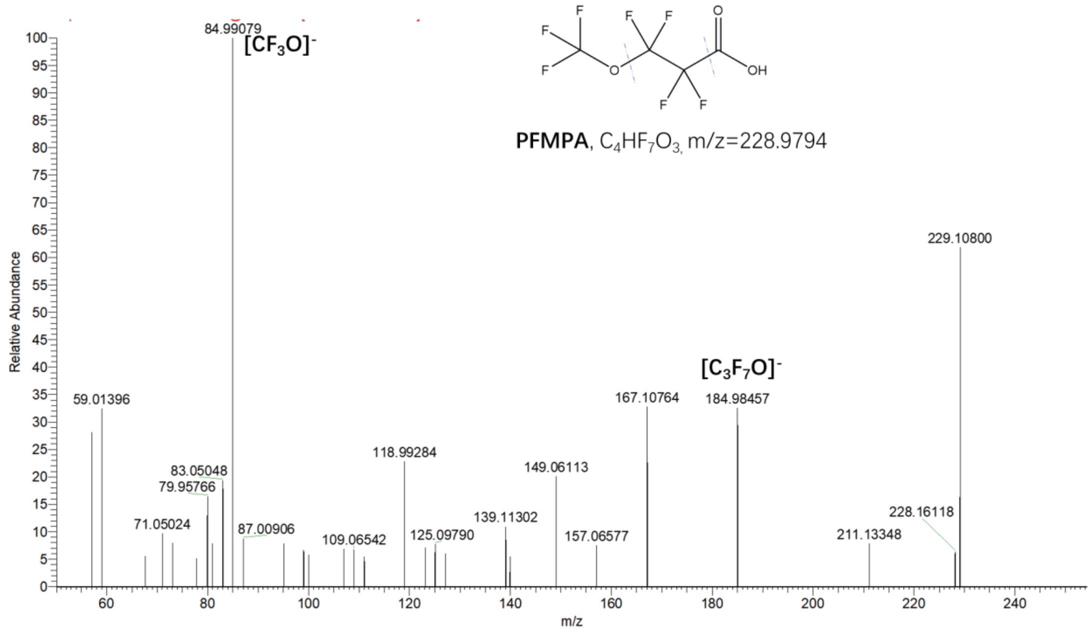

Figure S11 The MS2 spectra of PFMPA

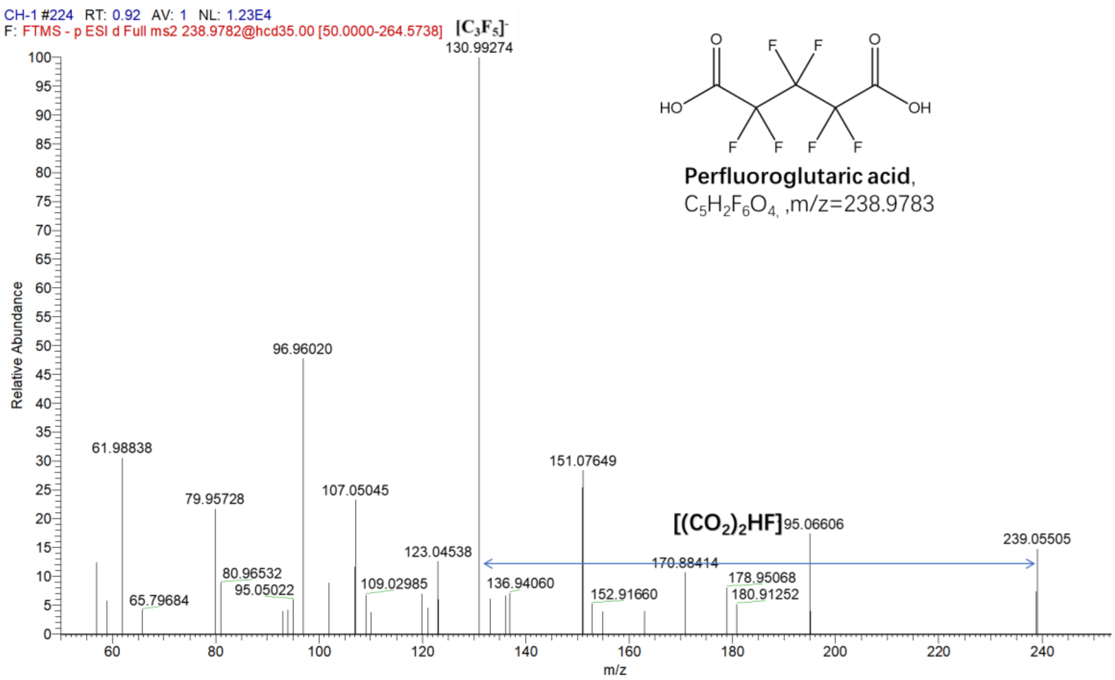

Figure S12 The MS2 spectra of PFGdiA

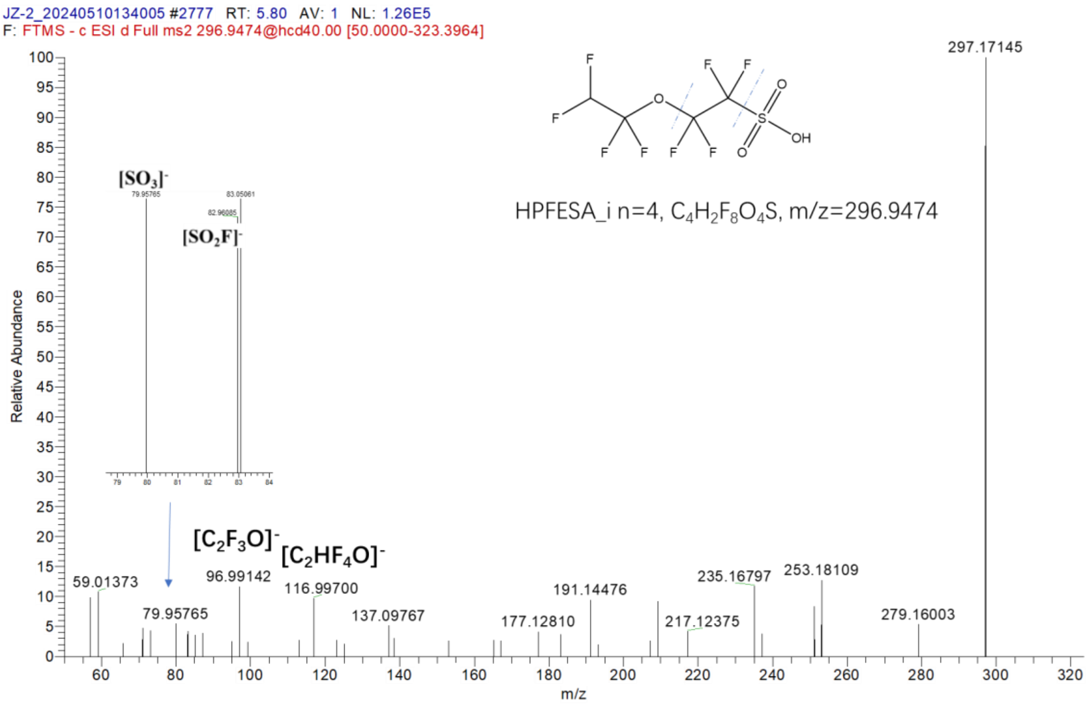

Figure S13 The MS2 spectra of HPFLSA_i n=4

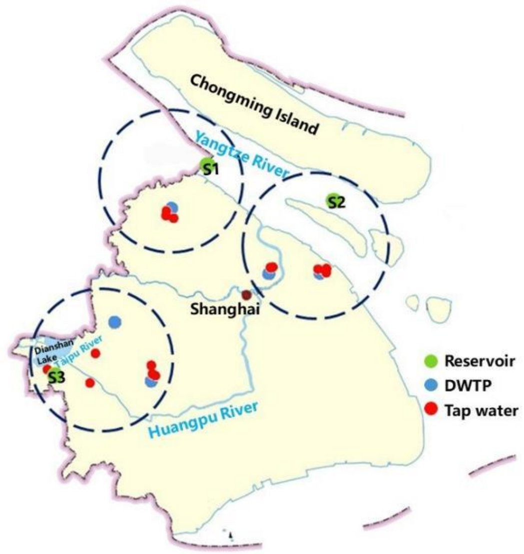

Figure S14 The Sampling point map

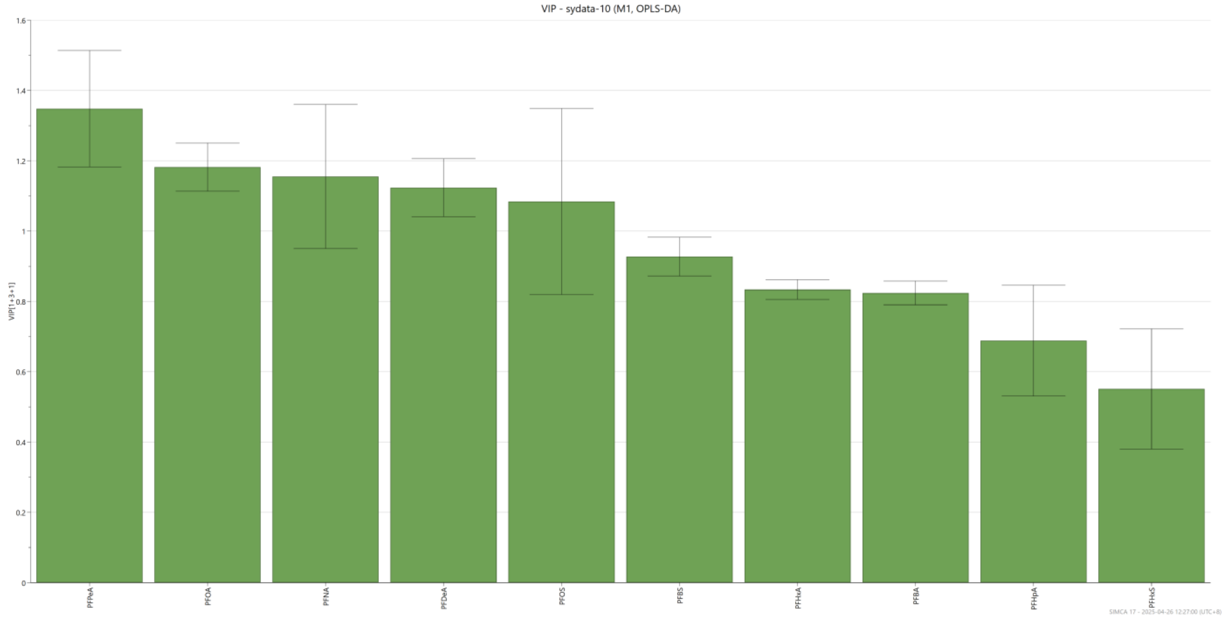

Figure S15 The VIP of variable in OPLS-DA
